# *In vivo* Assembly in *Escherichia coli* of Transformation Vectors for Plastid Genome Engineering

**DOI:** 10.3389/fpls.2017.01454

**Published:** 2017-08-21

**Authors:** Yuyong Wu, Lili You, Shengchun Li, Meiqi Ma, Mengting Wu, Lixin Ma, Ralph Bock, Ling Chang, Jiang Zhang

**Affiliations:** ^1^Hubei Collaborative Innovation Center for Green Transformation of Bio-Resources, College of Life Sciences, Hubei University Wuhan, China; ^2^Hubei Key Laboratory of Industrial Biotechnology, College of Life Sciences, Hubei University Wuhan, China; ^3^Department III, Max-Planck-Institut für Molekulare Pflanzenphysiologie Potsdam, Germany

**Keywords:** plastid transformation, transformation vector, vector assembly, iVEC, high-level expression, GFP

## Abstract

Plastid transformation for the expression of recombinant proteins and entire metabolic pathways has become a promising tool for plant biotechnology. However, large-scale application of this technology has been hindered by some technical bottlenecks, including lack of routine transformation protocols for agronomically important crop plants like rice or maize. Currently, there are no standard or commercial plastid transformation vectors available for the scientific community. Construction of a plastid transformation vector usually requires tedious and time-consuming cloning steps. In this study, we describe the adoption of an *in vivo Escherichia coli* cloning (iVEC) technology to quickly assemble a plastid transformation vector. The method enables simple and seamless build-up of a complete plastid transformation vector from five DNA fragments in a single step. The vector assembled for demonstration purposes contains an enhanced green fluorescent protein (GFP) expression cassette, in which the *gfp* transgene is driven by the tobacco plastid ribosomal RNA operon promoter fused to the 5′ untranslated region (UTR) from *gene10* of bacteriophage T7 and the transcript-stabilizing 3′UTR from the *E. coli* ribosomal RNA operon *rrnB*. Successful transformation of the tobacco plastid genome was verified by Southern blot analysis and seed assays. High-level expression of the GFP reporter in the transplastomic plants was visualized by confocal microscopy and Coomassie staining, and GFP accumulation was ~9% of the total soluble protein. The iVEC method represents a simple and efficient approach for construction of plastid transformation vector, and offers great potential for the assembly of increasingly complex vectors for synthetic biology applications in plastids.

## Introduction

Plastid transformation has become a promising approach for both basic research and plant biotechnology applications. Compared with conventional nuclear transformation, plastid transformation offers several distinct advantages, including high-level transgene expression, to up to 70% of the total soluble protein (TSP) (Oey et al., 2009), multi-gene engineering with operons (Zhou et al., 2008; Lu et al., 2013; Fuentes et al., 2016), maternal inheritance largely preventing transgene spread via pollen (Greiner et al., 2015), and absence of epigenetic effects thus avoiding gene silencing and position effects (Maliga, 2004; Bock, 2007, 2015; Maliga and Bock, 2011). However, only a handful of plant species (e.g., tobacco, tomato, lettuce) is currently amenable to plastid transformation, which severely constrains the application of this technology. Design and construction of suitable transformation vectors are inherent steps in plastid genome engineering. Since integration of foreign DNA into the plastid genome of seed plants is dependent upon homologous recombination (Svab et al., 1990), vector construction involves the incorporation of flanking sequences that are homologous to the target plastid genome. Moreover, identification of suitable combinations of the sequence elements involved in the control of plastid transgene expression (e.g., promoters, 5′ and 3′ untranslated regions) for optimized expression of the transgenes (i.e., adjustment of transgene expression to the desired level) usually requires construction of a set of different plastid transformation vectors and their test *in vivo*. The aforementioned cumbersome cloning procedures involved are becoming a limiting step in plastid genome engineering.

The design and generation of DNA constructs represents a major part of the work in all laboratories that use transgenic technologies. The traditional “cut-and-paste” method for vector construction, which largely relies on enzymatic digestion and ligation reactions using restriction enzymes and DNA ligase, has remained the standard approach for over 40 years. However, limited choices of restriction enzymes, problems with lowly efficient DNA digestion and/or ligation, and a sometimes high background of clones representing the religated vector lacking the insert DNA often cause difficulties in vector construction (Nakayama and Shimamoto, 2014). Site-specific recombination systems such as the Gateway™ (Karimi et al., 2002; Curtis and Grossniklaus, 2003) and Creator™ (Marsischky and LaBaer, 2004; Berrow et al., 2007) systems have been developed to replace the “cut-and-paste” approach. Also, all the methods discussed above usually leave behind unwanted sequences (e.g., restriction sites or site-specific recombination sequences) at the cloning junctions, which may interfere with faithful gene function.

A typical plastid transformation vector contains two flanking sequences with homology to the plastid genome of the target species. These flanks are required for site-specific integration of the transgene(s) into the recipient plastid genome by homologous recombination. In addition, the vector must contain a selectable marker gene cassette that functions in the selection of cells with transgenic plastids (referred to as transplastomic cells), and one or more transgene expression cassette(s). Many studies have shown that plastid gene and transgene expression is mainly regulated at the post-transcriptional level (Staub and Maliga, 1994; Eberhard et al., 2002; Bock, 2007; Kahlau and Bock, 2008; Valkov et al., 2009). The 5′ untranslated region (UTR) and especially the ribosome-binding site (Shine-Dalgarno sequence) and its distance to the translational start codon are crucial determinants of the expression level (Ye et al., 2001; Drechsel and Bock, 2011). The requirements for proper spacing necessitate seamless cloning of the coding region of the transgene downstream of the 5′UTR. Recently, a number of seamless gene fusion methods have been developed for plasmid construction (Lu, 2005; Motohashi, 2017). Some of these methods have become available as commercial kits such as the In-Fusion® Cloning System (Zhu et al., 2007; Sleight et al., 2010), the USER™ Cloning (Geu-Flores et al., 2007) and the Gibson Assembly® Cloning (Gibson et al., 2009), which however are rather costly. Alternatively, target DNA molecules and vector fragments that contain identical sequences at both termini can also be ligated by cellular extracts from various laboratory *E. coli* strains (Zhang et al., 2012b; Motohashi, 2015; Okegawa and Motohashi, 2015b), referred to as seamless ligation cloning extract (SLiCE), or by utilizing the endogenous homologous recombination activity of bacterial cells, a method dubbed *in vivo E. coli* cloning (iVEC) (Bubeck et al., 1993; Oliner et al., 1993; Zhu et al., 2010; Li et al., 2011; Jacobus and Gross, 2015; Motohashi, 2017). Both SLiCE and iVEC offer simple and low-cost seamless methods for DNA cloning that do not require recombinant enzymes. These distinct advantages have prompted us to explore the iVEC method for the construction of plastid transformation vectors and test its efficiency.

To this end, we simultaneously introduced four DNA fragments (two flanking regions for homologous recombination and two expression cassettes) and one linear vector backbone into *E. coli* cells via the iVEC method, generating a plastid transformation vector for expression of the model protein green fluorescence protein (GFP) from the tobacco plastid genome. Controlling the *gfp* reporter gene by the tobacco rRNA operon promoter combined with the 5′UTR from *gene10* of bacteriophage T7 (*NtPrrn:T7g10*) and the 3′UTR from the *E. coli rrnB* operon (*TrrnB*), we show that the transplastomic plants generated with the iVEC-assembled transformation vector efficiently express the transgene, reaching protein accumulation levels of 9% of the TSP.

## Materials and methods

### Vector construction for tobacco plastid transformation

**Figure 1:**
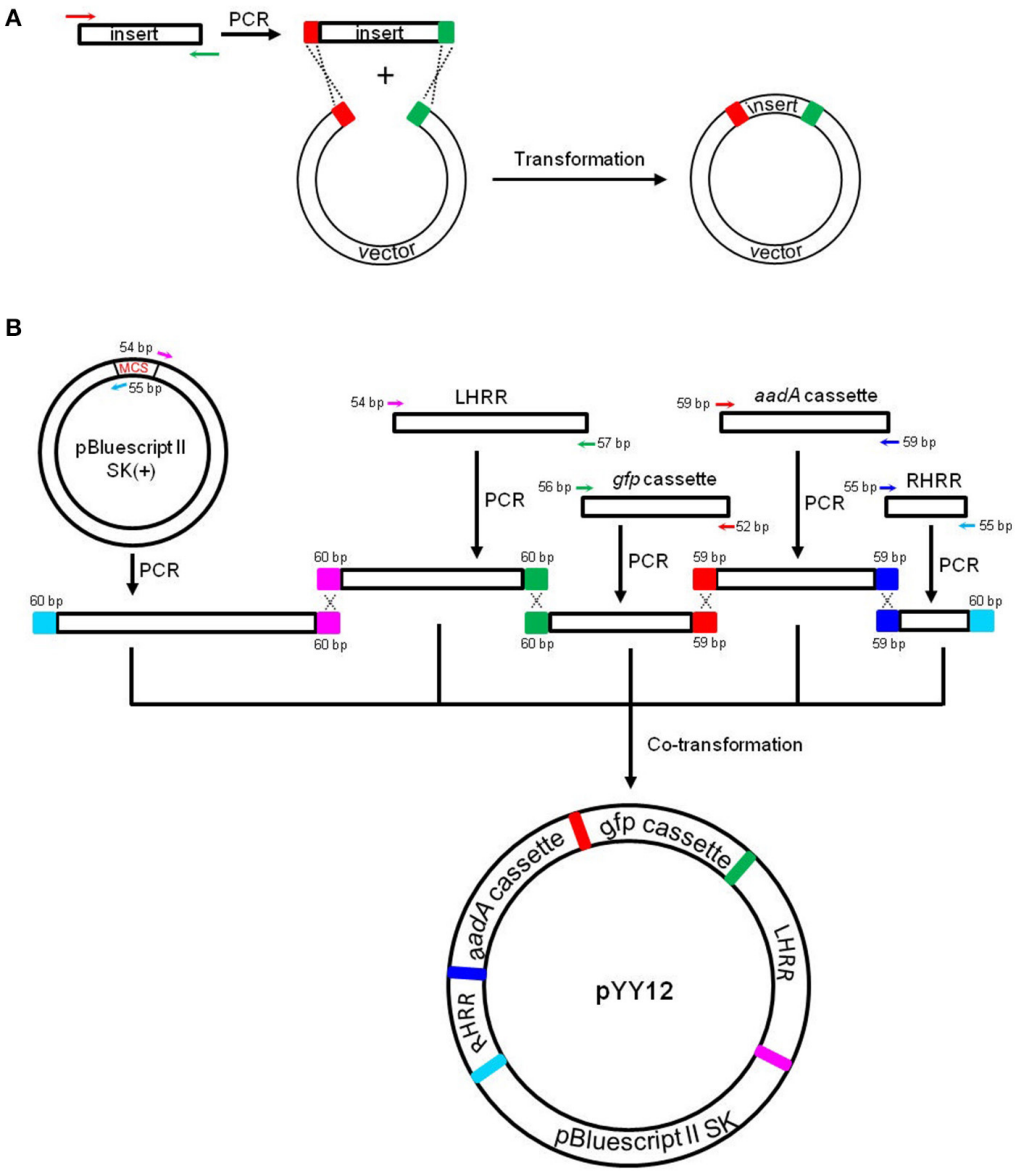
Principles of the *in vivo E. coli* cloning (iVEC) method and its application for the construction of plastid transformation vectors. **(A)** Schematic representation of iVEC illustrating integration of one insert into a cloning vector using the endogenous homologous recombination machinery of *E. coli*. **(B)** iVEC strategy for generation of plastid transformation vector pYY12. Five PCR products comprising the four DNA inserts (LHRR, RHRR, *aadA*, and *gfp* expression cassettes) and the linear backbone fragment (SK), pairwise sharing homologous sequences (59–60 bp) at their ends, were co-transformed into *E. coli*. LHRR, left homologous recombination region; RHRR, right homologous recombination region. Boxes of the same colors indicate the homologous sequences. Note short primer pairs with overlap at their 3' ends can generate PCR products with longer homologous end regions.

A tobacco plastid transformation vector was constructed based on the iVEC technology (Figure 1A). The vector mediates integration of the expression cassettes into the intergenic spacer region between the *trnfM* and *trnG* genes of the tobacco plastid genome. For vector assembly, we first amplified five DNA fragments which have pairwise homologous end sequences by Pfu DNA polymerase using a standard PCR protocol (95°C 3 min; 95°C 30 s, 55°C 30 s, 72°C 3 min, 30 cycles; 72°C 7 min). The two DNA fragments providing the flanking regions for homologous recombination, LHRR (containing *psaB, rps14* and *trnfM*, 1,940 bp) and RHRR (containing *psbZ* and *trnG*, 723 bp), were amplified from tobacco genomic DNA by PCR using primer pairs LHRR-F/LHRR-R and RHRR-F/RHRR-R, respectively. The primer sequences are listed in Table 1. Two additional DNA fragments were generated by PCR amplification with primer pairs aadA-F/aadA-R and gfp-F/gfp-R using *aadA* and *gfp* expression cassettes as templates that had been chemically synthesized (GeneCreate, China). The selectable marker gene *aadA* is driven by the *Chlamydomonas reinhardtii psbA* promoter (*CrPpsbA*), and followed by the 3′UTR from the *C. reinhardtii rbcL* gene (*CrTrbcL*). The cassette (1,647 bp) is flanked by two *loxP* sites to facilitate marker gene excision by Cre-mediated site-specific recombination (Corneille et al., 2001; Zhou et al., 2008). The reporter gene *gfp* is controlled by the tobacco plastid rRNA operon promoter combined with the 5′UTR from *gene10* of bacteriophage T7 (*NtPrrn:T7g10*) (Kuroda and Maliga, 2001), and the 3′UTR from the *E. coli* ribosomal RNA operon *rrnB* (*TrrnB*). *Nco*I and *Xba*I restriction sites were introduced at the 5′ and 3′ end, respectively, of the *gfp* gene (1,282 bp). The fifth DNA fragment (SK, 2,916 bp) was the vector backbone fragment from pBluescript II SK (+) without the multiple cloning site (MCS) amplified using primers pBS-F/pBS-R (Table 1). The five DNA fragments comprising the four DNA insert pieces (LHRR, 133 ng; RHRR, 50 ng; *aadA* expression cassette, 113 ng; *gfp* expression cassette, 88 ng) and the linear vector backbone fragment (100 ng) were mixed in a stoichiometric ratio of 2:2:2:2:1, and then co-transformed into *E. coli* (XL10-Gold, Agilent technologies) chemically competent cells (>2 × 10^8^ cfu/μg assayed on pUC19). Positive clones were identified by selection for both ampicillin and spectinomycin resistance and further confirmed by their green fluorescence and by DNA sequencing.

**Table 1:** Oligonucleotide sequences used in this study (homologous sequences are underlined).

**Primer**	**Sequence (5′ → 3′)**	**Primer length (bp)**	**PCR product end homology length (bp)**
LHRR-F	GTAATACGACTCACTATAGGGCGAATTGGAGCCTTGCTCTAGCTTCTTTAGGGG	54	60
LHRR-R	TCCAGCTTTTGTTCCCTTTAGTGAGGGTTAAACCCTAAAATAGTTTGGCAAAACAAG	57	60
RHRR-F	TTCGTATAGCATACATTATACGAAGTTATACTAGTAATTAATTCCCGCCTTTCGC	55	59
RHRR-R	TTAACCCTCACTAAAGGGAACAAAAGCTGGTGATTAGCGTACCCGTTGTATTTGC	55	60
aadA-F	GGATGGCCTTTTTGCGTTTCTACGGGCCCGGTACCATAACTTCGTATAGCATACATTAT	59	59
aadA-R	AAAGCGAAAGGCGGGAATTAATTACTAGTATAACTTCGTATAATGTATGCTATACGAAG	59	59
gfp-F	CATCTTGTTTTGCCAAACTATTTTAGGGTTTAACCCTCACTAAAGGGAACAAAAGC	56	60
gfp-R	ATAATGTATGCTATACGAAGTTATGGTACCGGGCCCGTAGAAACGCAAAAAG	52	59
pBS-F	GAGAAGCAAATACAACGGGTACGCTAATCACCAGCTTTTGTTCCCTTTAGTGAGG	55	60
pBS-R	TAATAACCCCTAAAGAAGCTAGAGCAAGGCTCCAATTCGCCCTATAGTGAGTCG	54	60
psaB-Fp	CCCAGAAAGAGGCTGGCCC	19	
psaB-Rp	CCCAAGGGGCGGGAACTGC	19	
gfp-Fp	ATGGTGAGTAAAGGAGAAGAACTTTTC	27	
gfp-Rp	TTACTTGTACAGCTCGTCCATGCC	24	

### Plant material and growth conditions

Tobacco seedlings (*Nicotiana tabacum* L. cv Petit Havana) were raised axenically on MS medium containing 3% (w/v) sucrose (Murashige and Skoog, 1962). Regenerated homoplasmic shoots from transplastomic *N. tabacum* lines were rooted and propagated on the same medium. Rooted plants were transferred to soil and grown in a controlled-environment greenhouse in 50–200 μE m^−2^ s^−1^ constant light under a 16 h light/8 h dark photoperiod, at 25°C/20°C and 55% humidity.

### Plastid transformation and selection of transplastomic lines

DNA for plastid transformation was prepared using the Nucleobond Xtra Plasmid Midi Kit (Macherey-Nagel, Germany). Young leaves from four-week old tobacco plants were bombarded with DNA-coated 0.6 μm gold particles using a PDS-1000/He Biolistic Particle Delivery System (Bio-Rad). The bombarded leaf samples were cut into 5 × 5 mm squares and selected on RMOP medium containing 500 mg/L spectinomycin (Svab and Maliga, 1993). In general, spectinomycin-resistant shoots appeared within 1 month. The initial shoots were further screened by double resistance tests on medium supplemented with 500 mg/L spectinomycin and 500 mg/L streptomycin to eliminate spontaneous spectinomycin-resistant mutants (Bock, 2001). Several independent transplastomic lines were obtained and subjected to two additional rounds of regeneration on RMOP/spectinomycin medium to select for homoplasmy. Homoplasmic shoots were transferred onto rooting medium (MS containing 0.1 mg/L NAA and 500 mg/L spectinomycin). Rooted shoots were transferred to soil and grown to maturity in the greenhouse.

### DNA extraction and southern blot analysis

Total plant DNA was isolated from leaves of wild type and spectinomycin-resistant plants using a cetyltrimethylammonium bromide-based extraction method (Doyle and Doyle, 1990). DNA samples (5 μg total cellular DNA) were digested with *Bgl*II for 16 h, separated by electrophoresis in 1% agarose gels and transferred onto a positively charged nylon membranes (GE Healthcare, USA) by capillary action using the semi-dry transfer method. A 550 bp fragment of the *psaB* gene was amplified by PCR from tobacco plastid DNA using primer pair psaB-Fp/psaB-Rp (Table 1), and used as hybridization probe to verify plastid transformation. Labeling of the probe and hybridization were performed with the DIG-High Prime DNA Labeling and Detection Starter Kit II following the manufacturer's instructions (Roche, USA).

### RNA isolation and northern blot analysis

Total RNA was isolated from fresh leaves of wild type and transplastomic plants using the Trizol Reagent (Invitrogen, USA) and following the manufacturer's instruction. RNA samples (3 μg total RNA) were denatured and separated by electrophoresis in formaldehyde-containing 1% agarose gels. The separated RNA molecules were then transferred from the gel to a positively charged nylon membrane (GE Healthcare, USA). The *gfp* probe, a 720-bp fragment, was amplified by PCR from plasmid pYY12 (Figure 1) with primer pair gfp-Fp/gfp-Rp. The probe was labeled with DIG using the PCR DIG probe synthesis kit following the manufacturer's protocol (Roche, USA). RNA blots were hybridized at 42°C.

### Protein extraction and GFP quantification by SDS-PAGE

Leave samples (~100 mg) of wild type and transplastomic tobacco plants were ground in liquid nitrogen and total protein was isolated using a phenol-based extraction method (Cahoon et al., 1992). Protein concentrations were measured with the Easy II Protein Quantitative Kit (TransGen Biotech, China). Samples of 5 μg total protein were separated by SDS-PAGE (18% gels), and the proteins were visualized by Coomassie Brilliant Blue R-250 staining. Recombinant GFP (rGFP, Vector Laboratories, UK) was loaded in different amounts for semi-quantitative analysis.

### Detection of GFP by fluorescence microscopy

For fluorescence microscopy, thin sections of leaves (comprising the epidermis and part of the mesophyll) were mounted in water under glass coverslips. GFP and chlorophyll fluorescences were analyzed by laser-scanning confocal microscopy (LSM 710, Carl ZEISS). For GFP detection, a wavelength of 488 nm and a filter of 500–530 nm were used for excitation and signal analysis, respectively. The autofluorescence of chlorophyll was analyzed with a 488 nm argon laser in combination with a 688–757 nm filter set.

## Results

### Construction of plastid transformation vector

For fast and straightforward construction of a plastid transformation vector, we employed the iVEC cloning technology. The general principle of iVEC is illustrated for insertion of a single fragment in Figure 1A. The mixture of PCR-amplified insert and linearized vector, which have homologous sequences at both of their ends (red and green color, respectively, in Figure 1A), are directly co-transformed into *E. coli* competent cells. The insert then recombines into the vector *in vivo*, owing to the homologous recombination activity present in *E. coli*. To assembly a plastid transformation vector by iVEC, four DNA fragments (LHRR, RHRR, *aadA* and *gfp* cassettes) and the pBluescript SK (+) backbone without the MCS were amplified using specific primer pairs that harbor appropriate substrate sequences (59–60 bp) for homologous recombination (Table 1). The five resulting DNA fragments were then mixed and co-introduced into *E. coli* (XL10-Gold) competent cells to generate plasmid pYY12 (Figure 1B). Typically, all ampicillin and spectinomycin-resistant clones that were obtained showed green fluorescence (Figure S1A), indicating presence of the *gfp* reporter gene. The plasmids isolated from 12 independent clones were verified by restriction enzyme digestion (Figure S1B) and confirmed by DNA sequencing. The sequencing results revealed 100% correctly assembled clones with precisely fused fragments, although the value maybe an overestimate due to selection on medium containing both ampicillin and spectinomycin, a procedure that might eliminate possible false positive clones.

### Generation of transplastomic tobacco lines

**Figure 2:**
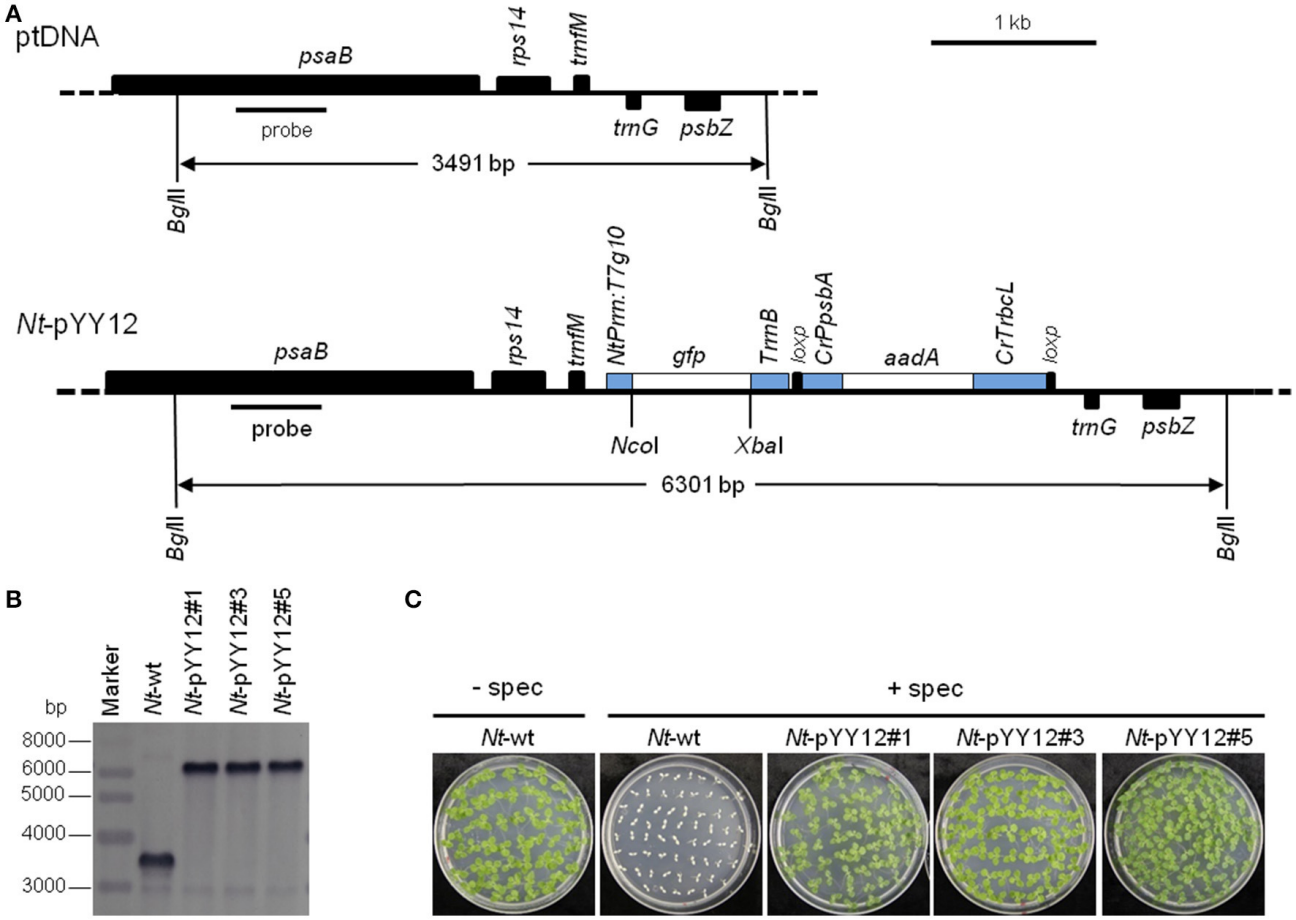
Introduction of a *gfp* reporter gene into the tobacco plastid genome with an iVEC-produced transformation vector. **(A)** Physical map of the plastid genome region (ptDNA) used for integration of the *gfp* cassette and maps of the transgenic loci in the generated transplastomic tobacco lines (*Nt*-pYY12). The *Bgl*II restriction sites used in Southern blot analysis and the expected fragment sizes are indicated. The location of the hybridization probe is shown as a black bar. *Cr*: *C. reinhardtii*; *Nt*: *N. tabacum*; T7: bacteriophage T7; P: promoter; T: terminator. **(B)** Southern blot analysis of transplastomic plants. Three independently isolated transplastomic lines were analyzed. Total DNA was digested with *Bgl*II, and hydrized to a DIG-labeled probe shown in **(A)**. The absence of a hybridization signal for the wild-type genome indicates homoplasmy of all transplastomic lines. **(C)** Seed germination assays on medium containing 500 mg/L spectinomycin confirm the homoplasmic state of the transplastomic lines. Germination of wild-type seeds on antibiotic-free medium (*Nt*-wt) was included as positive control.

**Figure 3:**
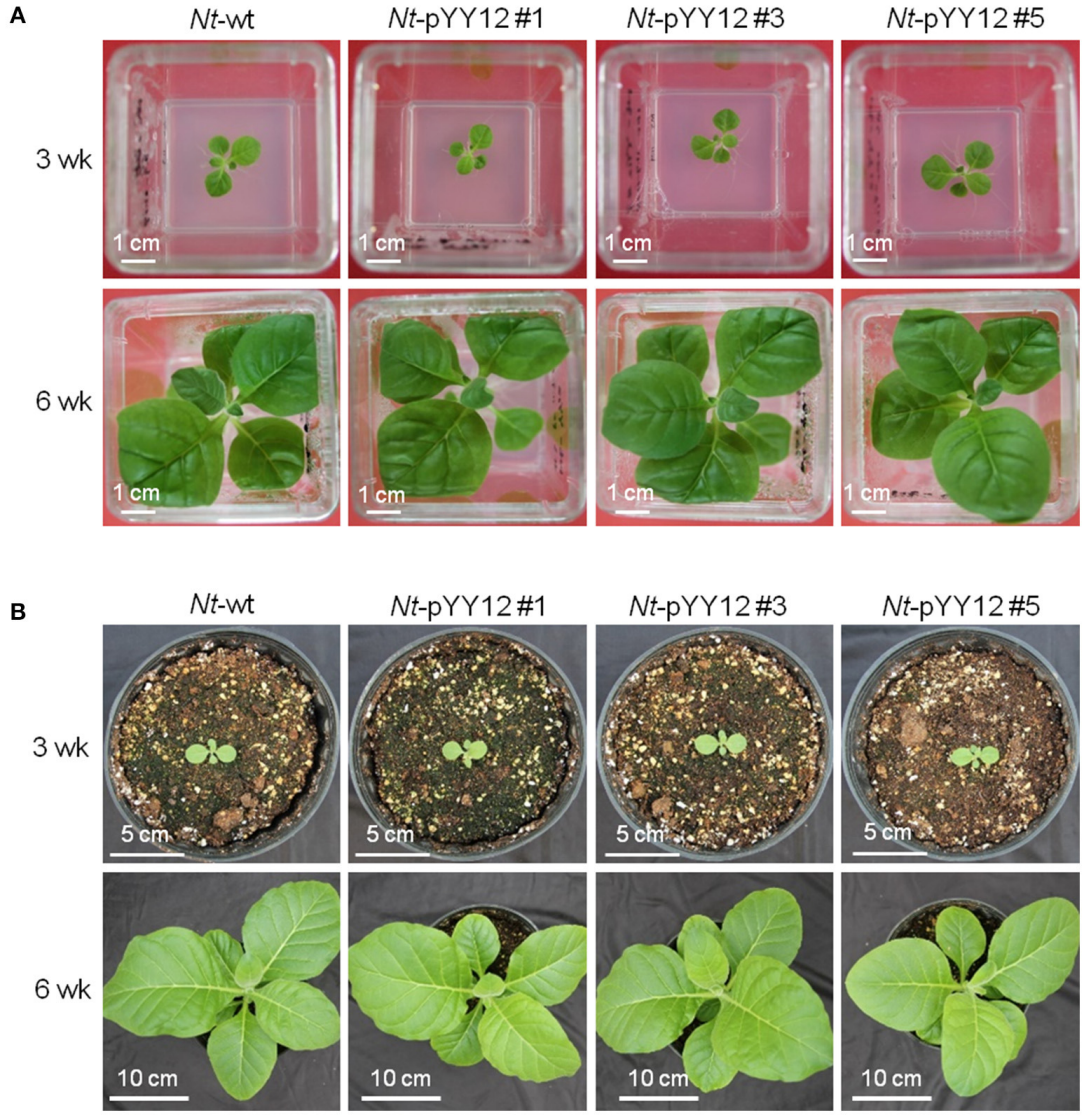
Phenotypes of wild type and transplastomic plants. Plants are shown after **(A)** 3 weeks (upper panel) and 6 weeks (lower panel) of growth under heterotrophic conditions in sterile culture on sucrose-containing synthetic medium, **(B)** 3 weeks (upper panel) and 6 weeks (lower panel) of growth under photoautotrophic conditions in soil.

To confirm the suitability of the iVEC-assembled vector for plastid transformation, vector pYY12 was introduced into tobacco plastids by particle bombardment and transgenic shoots were regenerated on a synthetic plant regeneration medium containing spectinomycin (Svab and Maliga, 1993). A number of transformed plant lines were obtained and subjected to two or three additional regeneration rounds on spectinomycin-containing medium. Three independently transformed plant lines (*Nt*-pYY12#1, *Nt*-pYY12#3 and *Nt*-pYY12#5) were further characterized. Southern blot analysis verified the homoplasmic state of the *Nt*-pYY12 lines, as evidenced by absence of a hybridization signal for the 3,491 bp fragment diagnostic of the wild type and exclusive presence of the 6,301 bp fragment expected in transplastomic plants (Figures 2A,B). To further support this conclusion, seed assays were conducted. The progenies of the *Nt*-pYY12 lines showed no segregation on spectinomycin-containing medium (Figure 2C), which is generally considered the ultimate proof of homoplasmy of transplastomic lines. Transplastomic *Nt*-pYY12 lines grew normally and were indistinguishable from wild-type plants under both heterotrophic conditions (i.e., upon growth in sucrose-containing synthetic medium) and autotrophic growth conditions (i.e., growth in soil in the greenhouse; Figure 3).

### Determination of *Gfp* expression levels in transplastomic plants

**Figure 4:**
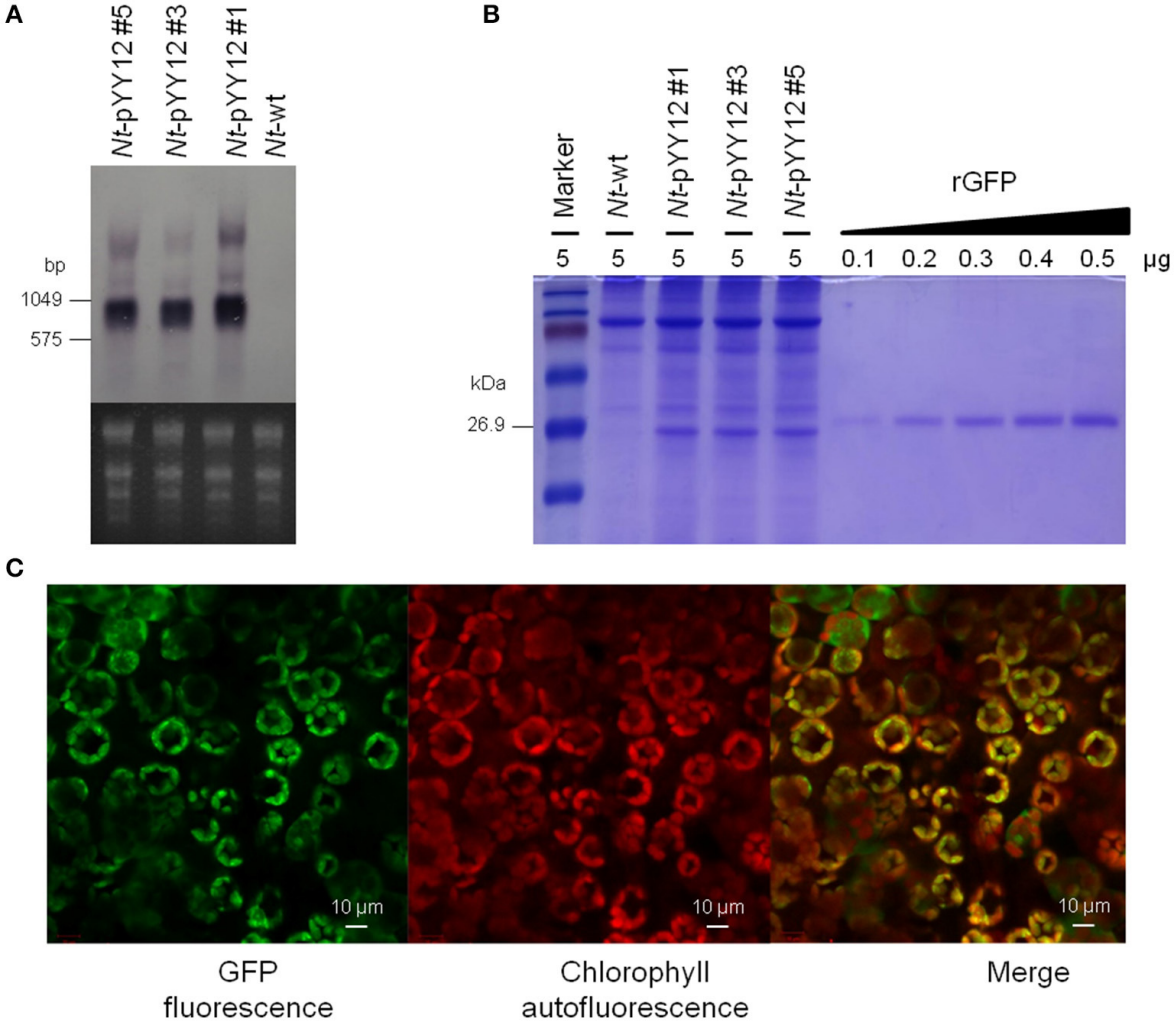
Analysis of GFP expression in transplastomic tobacco lines. **(A)** Northern blot analysis of *gfp* mRNA accumulation in leaves of transplastomic tobacco plants. The ethidium bromide-stained gel prior to blotting is shown below the blot. **(B)** GFP accumulation in leaves. Protein samples were separated by 18% SDS-PAGE and the gel was subsequently stained by Coomassie Brilliant Blue R-250. The amount of total protein loaded in each lane is indicated (μg). For semi-quantitative analysis, a dilution series of recombinant GFP was loaded. Note the larger size of the rGFP due to its hexahistidine tag. **(C)** Subcellular localization of GFP in transplastomic plants. The images correspond to subepidermal cells of 8-week-old seedlings. From left to right: GFP fluorescence (green), chlorophyll autofluorescence (red), and merged images.

To examine *gfp* transcript accumulation, northern blot experiments were performed using a hybridization probe specific for the *gfp* coding region. Hybridization to the *gfp* probe detected three transcripts (Figure 4A), with the smallest and by far most abundant transcript of ~1,000 nt representing the expected full-length *gfp* mRNA. The lowly abundant larger bands most probably represent read-through transcripts, which are common in plastids (Zhou et al., 2008; Elghabi et al., 2011) and usually originate from inefficient transcription termination.

The expression level of GFP produced by the transplastomic lines was determined in a semiquantitative manner by comparison with a dilution series of recombinant GFP. GFP amounts were estimated to reach more than 9% of the TSP (Figure 4B). GFP fluorescence signal in chloroplasts and chloroplast autofluorescence (i.e., red chlorophyll fluorescence) were monitored by confocal laser-scanning microscopy. As expected, the green GFP fluorescence was co-localized with the red autofluorescence of the chloroplasts in leaves of the transplastomic plants (Figure 4C), well in line with GFP synthesis within the chloroplast compartment.

## Discussion

Traditional molecular cloning depends on restriction endonucleases to produce linear vectors and inserts that can be fused by T4 DNA ligase. However, this “cut-and-paste” method can be inefficient and time-consuming, due to stepwise construct assembly. Moreover, conventional cloning methods require careful selection of suitable restriction endonucleases and often either necessitates prior introduction of suitable restriction sites by site-directed mutagenesis or leads to incorporation of unwanted nucleotide sequences (due to limited choices of restriction sites). The latter can result in undesired changes, for example by changing the spacing between regulatory sequences (e.g., translation initiation signals) and the coding region. Recently, two strategies for modular cloning of plastid vectors have been developed. While one of the methods uses the Gateway system to simplify vector construction (Gottschamel et al., 2013), the other employs modular combination of genetic elements with type II restriction endonucleases (GoldenBraid) to construct chloroplast vectors (Vafaee et al., 2014).

Although making plastid transformation vector construction easier, both methods require expensive commercial enzymes and/or kits. The iVEC method described in this study offers a cost-effective and conceptually simpler approach for the construction of plastid transformation vectors. Most importantly, vector assembly can be done in a single step, as demonstrated here for our new plastid transformation vector pYY12 (Figure 1). We have shown that assembly of four inserts and a linearized vector DNA fragments via iVEC is readily possible. Assembly from five fragments would be the standard approach for construction of plastid transformation vectors that express a single transgene of interest in addition to the selectable marker gene. The approach also can be easily adopted to construct plastid transformation vectors for new plant species that are not yet amenable to plastid engineering (our unpublished results). In addition, since the *gfp* gene in pYY12 is flanked by two restriction site (*Nco*I and *Xba*I), any other gene of interest can be readily cloned into pYY12 through either traditional “cut-and-paste” or the iVEC method. Although iVEC has a lower transformation efficiency due to the requirement for co-transformation when compared with other *in vitro* seamless cloning methods such as SLiCE and In-Fusion (Okegawa and Motohashi, 2015a,b), it should not pose a serious bottleneck if highly competent *E. coli* cells and/or efficient transformation methods such as electroporation are used. In addition, longer homologous regions (30–50 bp) can increase the cloning efficiency of the iVEC method (Kostylev et al., 2015; Motohashi, 2017). Therefore, we applied longer (59–60 bp) homologous end regions to assemble plastid transformation vector pYY12 by iVEC. Moreover, we have shown that the cloning efficiency obtained with the iVEC method is reproducible and reliable (Table S1).

Plastids are descendants of formerly free-living cyanobacteria and have retained many prokaryotic features. Because plastid gene expression is predominantly regulated at the post-transcriptional level, especially at the translational level (Stern et al., 2010; Drechsel and Bock, 2011), the choice of the 5′UTR, often harboring a Shine-Dalgarno-like sequence, is of particular importance (Eberhard et al., 2002; Drechsel and Bock, 2011) in determining the expression level of a given transgene. The 5′UTR from *gene10* of bacteriophage T7 proved to be superior to all plastid 5′UTRs and confers extreme transgene expression levels of up to 70% of the TSP in plastid (Kuroda and Maliga, 2001; Ye et al., 2001; Oey et al., 2009). The 3′UTRs of plastid mRNAs are thought to function in transcript stability, by folding into a stable stem-loop-type RNA secondary structure (Stern and Gruissem, 1987) that blocks exoribonucleolytic degradation. A large number of plastid promoters and UTRs conferring different strengths of transgene expression have been described (Tangphatsornruang et al., 2011; Zhang et al., 2012a; Bock, 2015). In our plastid transformation vector pYY12, we have chosen the most commonly used strong promoter (the 16S ribosomal RNA operon promoter, *Prrn*), the strongest known translation signal (the *gene10* leader from bacteriophage T7, *T7g10*), and the 3′UTR of the ribosomal RNA operon *rrnB* from *E. coli* (*TrrnB*) that confers maximum transcript stability (Tangphatsornruang et al., 2011). By combining these highly efficient regulatory elements, expression of GFP in our transplastomic plants reached very high levels of ~9% of TSP—a value significantly higher than in most previous studies on GFP expression in tobacco plastids (Sidorov et al., 1999; Reed et al., 2001; Newell et al., 2003; Gottschamel et al., 2013). GFP accumulated to only 3% of TSP in plastids when the same combination of promoter (*Prrn*) and 5′UTR (*T7g10*) was used (Gottschamel et al., 2013). Although it is believed that the 3′UTR does not strongly affect translation efficiency (Tangphatsornruang et al., 2011), it is possible that the increased transcript stability conferred by the superior properties of the *E. coli TrrnB* contribute to the higher GFP expression level in our lines. Alternatively, other differences between the two constructs, including, for example, the different integration sites in the plastid genome could also be causally responsible for the difference in GFP accumulation levels. In spite of the very high GFP accumulation of 9% of TSP in the *Nt*-pYY12 lines, recombinant protein expression did not have an apparent deleterious effect on plant growth or development (Figure 3), indicating that high levels of GFP are tolerated by the plastid and do not interfere with photosynthesis or gene expression.

## Author contributions

YW, LM, and JZ designed the experiments. YW, LY, MM, MW, and LC performed the experiments; SL, RB, and JZ analyzed the data and wrote the paper.

### Conflict of interest statement

The authors declare that the research was conducted in the absence of any commercial or financial relationships that could be construed as a potential conflict of interest.

## Supplementary material

Figure S1 Screening of recombinant clones. **(A)** The four inserts (LHRR, RHRR, *aadA*, and *gfp* cassettes) and the vector backbone from pBluescriptII SK (+) were co-transformed into *E. coli*, and the arrows indicated 19 *E. coli* clones growing on a selection plate that all display green GFP fluorescence under blue light. **(B)** Verification of 12 recombinant plasmids by *Kpn*I digestion and analysis by gel electrophoresis in a 1% agarose gel.

Table S1 Cloning efficiency of iVEC for the construction of plastid transformation vector pYY12.
