# Supplementary figures and images for: In vivo Assembly in Escherichia coli of Transformation Vectors for Plastid Genome Engineering
Source: Front Plant Sci. 2017 Aug 21;8:1454. doi: 10.3389/fpls.2017.01454 (PMC5566966; doi:10.3389/fpls.2017.01454)

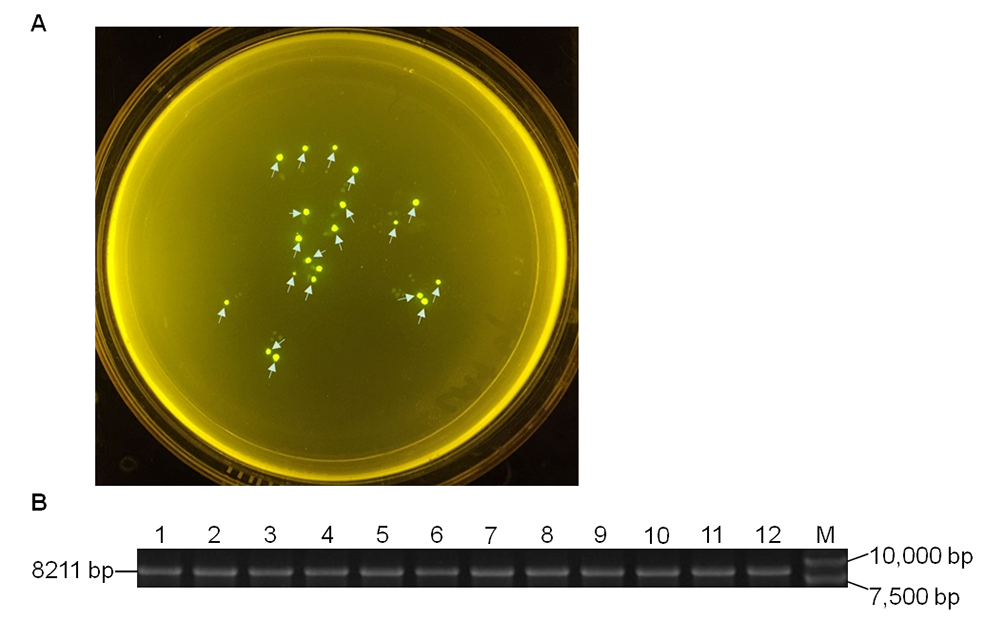

Supplement: Figure S1 — Screening of recombinant clones. (A) The four inserts (LHRR, RHRR, aadA, and gfp cassettes) and the vector backbone from pBluescriptII SK (+) were co-transformed into E. coli, and the arrows indicated 19 E. coli clones growing on a selection plate that all display green GFP fluorescence under blue light. (B) Verification of 12 recombinant plasmids by KpnI digestion and analysis by gel electrophoresis in a 1% agarose gel. [file Image1.TIF]
