# Supplementary material for: In vivo Assembly in Escherichia coli of Transformation Vectors for Plastid Genome Engineering
Source: Front Plant Sci. 2017 Aug 21;8:1454. doi: 10.3389/fpls.2017.01454 (PMC5566966; doi:10.3389/fpls.2017.01454)
Supplement: Table S1 — Cloning efficiency of iVEC for the construction of plastid transformation vector pYY12. [file Table1.DOCX]

**Table S1.** Cloning efficiency of iVEC for the construction of plastid transformation vector pYY12^a^

| Exp. No. | Number of colonies | Number of colonies with green fluorescence | Number of positive colonies^b^ | Cloning efficiency^c^ |
| --- | --- | --- | --- | --- |
| 1 | 15 | 15 | 15 | 100% |
| 2 | 12 | 12 | 12 | 100% |
| 3 | 6 | 6 | 6 | 100% |

^a^ Four insert DNA fragments (LHRR, RHRR, *aadA* expression cassette, *gfp* expression cassette) and linearized vector DNA were amplified by PCR, and purified by agarose gel electrophoresis and a Gel/PCR Extract Kit (Omega). The purified four insert DNA fragments (LHRR, 133 ng; RHRR, 50 ng; *aadA* expression cassette, 113 ng ; *gfp* expression cassette, 88 ng) and the linearized vector DNA (100 ng) were mixed in a stoichiometric ratio of 2:2:2:2:1, and then co-transformed into *E. coli* (XL10-Gold, Agilent technologies) chemically competent cells (1.05×10^8^ cfu/μg assayed on pUC19). Positive clones were identified by selection for both ampicillin and spectinomycin resistance.

^b^ Colony verification by *Kpn*I digestion (unique site at vector pYY12) and DNA sequencing. Note that the number of positive colonies slight decreases from experiment 1 to experiment 3, most likely due to the decrease over time in competence of the bacterial cells used for transformation.

^c^ Cloning efficiency is defined as the fraction of clones confirmed as correct by DNA sequencing compared to all green fluorescence positive clones.
